# Supplementary material for: Metropolitan age-specific mortality trends at borough and neighborhood level: The case of Mexico City
Source: PLoS One. 2021 Jan 19;16(1):e0244384. doi: 10.1371/journal.pone.0244384 (PMC7815139; doi:10.1371/journal.pone.0244384)

S1 - Metropolitan age-specific mortality trends at borough and neighbourhood level: The case of Mexico City

Karol Baca-López[1,2¶], Cristóbal Fresno[3¶], Jesús Espinal-Enríquez[2], Miriam V. Flores-Merino[4], Miguel A. Camacho-López[1], Enrique Hernández-Lemus[2,5*]

[1] School of Medicine, Autonomous University of the State of Mexico, Toluca, State of Mexico, Mexico
[2] Computational Genomics Department, National Institute of Genomic Medicine, Mexico City, Mexico
[3] Technology Development Department, National Institute of Genomic Medicine, Mexico City, Mexico
[4] School of Chemistry, Autonomous University of the State of Mexico, Toluca, State of Mexico, Mexico
[5] Centro de Ciencias de la Complejidad, Universidad Nacional Autonoma de Mexico, Mexico City, Mexico

* Corresponding author
E-mail: ehernandez@inmegen.gob.mx (EHL)
¶These authors contributed equally to this work.

1 Supplementary tables

1.1 Initial parameters

Table 1. Initial variogram parameter values for age-specific mortality rates.

		Time	Space	Joint		Time	Space	Joint
Infant					Pre-school			
	nugget	13.02	21.31	17.16		0.02	0.03	0.03
	sill	53.59	28.40	40.99		0.04	0.03	0.03
	range	12.00	13.27	12.64		12.00	13.27	12.64
	stAni	0.00	0.00	0.00		0.02	0.02	0.02
School					Productive			
	nugget	0.01	0.01	0.01		0.23	0.12	0.17
	sill	0.01	0.01	0.01		0.37	0.34	0.36
	range	12.00	13.27	12.64		12.00	13.27	12.64
	stAni	0.00	0.00	0.00		0.01	0.01	0.01
Post-produtive								
	nugget	9.03	10.58	9.81				
	sill	20.77	18.16	19.46				
	range	12.00	13.27	12.64				
	stAni	0.00	0.00	0.00				

stAni: spatio-temporal anisotropy

1.2 Adjusted models

Table 2. wMSE for the tested covariance models.

Model	Joint	Variogram model type: temporal + spatial								
		**Exp+Exp**	**Sph+Sph**	**Gau+Gau**	**Exp+Gau**	**Exp+Sph**	**Gau+Exp**	**Gau+Sph**	**Sph+Exp**	**Sph+Gau**
**Post-productive**										
metric		25.691566	16.987806	18.796475						
separable		40.959921	40.915431	40.930133	40.959921	40.915431	40.930133	40.959921	40.915431	40.930133
productSum		29.736305	29.107455	29.633025	29.736644	29.108073	29.495517	29.736644	29.108073	29.495517
sumMetric	Exp	18.325273	22.182019	17.305102	18.325226	21.494027	17.305133	18.325226	21.494027	17.305133
	Gau	16.935569	17.571166	15.203131	16.935461	15.709129	15.203128	16.935461	15.709129	15.203128
	Sph	23.046362	23.992436	24.340164	23.046361	23.992437	24.340164	23.046361	23.992437	24.340164
simpleSumMetric	Exp	15.764308	15.991394	16.521146	15.764307	15.991394	16.521143	15.764307	15.991394	16.521143
	Gau	14.660979	27.347296	**14.261569**	14.660979	27.347296	14.261572	14.660979	27.347296	14.261572
	Sph	27.282252	30.356947	27.853282	27.282252	30.356947	27.853282	27.282252	30.356947	27.853282
**Productive** ($x10^{-2}$)										
metric		8.6207099	8.8922886	8.7759266						
separable		9.2082780	9.2083721	9.2341280	9.2082780	9.2083721	9.2341280	9.2082780	9.2083721	9.2341280
productSum		8.5481668	8.5486867	8.5483792	8.5481668	8.5486867	8.5483792	8.5481668	8.5486867	8.5483792
sumMetric	Exp	7.6342080	8.5485855	8.2338415	**7.6333922**	8.5485855	8.2338415	7.6333922	8.5485855	8.2338415
	Gau	8.3531362	8.3091444	8.2999691	8.3531362	8.3091444	8.2999690	8.3531362	8.3091444	8.2999690
	Sph	8.5470949	8.5487401	8.5473143	8.5470949	8.5487401	8.5473143	8.5470949	8.5487401	8.5473143
simpleSumMetric	Exp	8.6220039	8.6239591	8.6532618	8.6220039	8.6239591	8.6532618	8.6220039	8.6239591	8.6532618
	Gau	8.8014332	8.7716488	8.7718362	8.8014332	8.7716488	8.7718362	8.8014332	8.7716488	8.7718362
	Sph	8.9242711	8.9551080	8.9085793	8.9242711	8.9551080	8.9085793	8.9242711	8.9551080	8.9085793

Table 2. wMSE for the tested covariance models.

		Variogram model type: temporal + spatial								
**Model**	**Joint**	**Exp+Exp**	**Sph+Sph**	**Gau+Gau**	**Exp+Gau**	**Exp+Sph**	**Gau+Exp**	**Gau+Sph**	**Sph+Exp**	**Sph+Gau**
					**School** ($x10^{-6}$)					
metric		**2.2939884**	2.3710215	2.3025265						
separable		2.2970406	2.2993204	2.3032552	2.2970406	2.2993204	2.3032552	2.2970406	2.2993204	2.3032552
productSum		3.3116894	3.4169090	3.3529686	3.3116894	3.4169090	3.3529686	3.3116894	3.4169090	3.3529686
sumMetric	Exp	2.8822070	3.3461985	3.0649730	2.8822070	3.3461985	3.0649730	2.8822070	3.3461985	3.0649730
	Gau	3.1318833	3.2362881	2.9491849	3.1318833	3.2362881	2.9491849	3.1318833	3.2362881	2.9491849
	Sph	2.8717123	2.9423249	2.8713554	2.8717123	2.9423249	2.8713554	2.8717123	2.9423249	2.8713554
simpleSumMetric	Exp	2.6529067	2.7649662	2.8198599	2.6529067	2.7649662	2.8198599	2.6529067	2.7649662	2.8198599
	Gau	2.8050087	2.6329603	2.5920438	2.8050087	2.6329603	2.5920438	2.8050087	2.6329603	2.5920438
	Sph	2.4813567	2.8219773	2.7436039	2.4813567	2.8219773	2.7436039	2.4813567	2.8219773	2.7436039
					**Pre-school** ($x10^{-5}$)					
metric		**5.3871475**	6.4569342	5.6881438						
separable		6.7138056	6.7234295	6.8297716	6.7138056	6.7234295	6.8297716	6.7138056	6.7234295	6.8297716
productSum		8.3793946	8.3626384	8.4044303	8.3793946	8.3626384	8.4044303	8.3793946	8.3626384	8.4044303
sumMetric	Exp	5.4396971	5.4393540	5.4770666	5.4396971	5.4393540	5.4770666	5.4396971	5.4393540	5.4770666
	Gau	5.7304361	5.7322290	5.7700965	5.7304361	5.7322290	5.7700965	5.7304361	5.7322290	5.7700965
	Sph	6.3862806	6.4159186	6.4315148	6.3862806	6.4159186	6.4315148	6.3862806	6.4159186	6.4315148
simpleSumMetric	Exp	5.4138608	5.4150930	5.4470521	5.4138608	5.4150930	5.4470521	5.4138608	5.4150930	5.4470521
	Gau	5.7102259	5.7156773	5.7444414	5.7102259	5.7156773	5.7444414	5.7102259	5.7156773	5.7444414
	Sph	6.6455263	6.3675970	6.4072491	6.6455263	6.3675970	6.4072491	6.6455263	6.3675970	6.4072491

Table 2. wMSE for the tested covariance models.

		Variogram model type: temporal + spatial								
**Model**	**Joint**	**Exp+Exp**	**Sph+Sph**	**Gau+Gau**	**Exp+Gau**	**Exp+Sph**	**Gau+Exp**	**Gau+Sph**	**Sph+Exp**	**Sph+Gau**
					**Infant**					
metric		374.8430	232.6356	358.6417						
separable		314.3353	314.3352	314.3353	314.3353	314.3352	314.3353	314.3353	314.3352	314.3353
productSum		309.4312	308.8565	308.1254	309.4312	308.8565	308.1254	309.4312	308.8565	308.1254
sumMetric	Exp	127.7998	131.6038	114.1761	118.1797	108.3609	101.2647	118.1797	108.3609	101.2647
	Gau	84.1927	84.2396	**82.8559**	85.0690	83.4854	82.9303	85.0690	83.4854	82.9303
	Sph	128.9818	115.4067	101.4309	128.9891	115.4161	101.4154	128.9891	115.4161	101.4154
simpleSumMetric	Exp	121.4418	108.5406	108.3578	121.4419	108.5406	108.3578	121.4419	108.5406	108.3578
	Gau	88.9448	113.7449	94.8973	88.9453	113.7446	94.8909	88.9453	113.7446	94.8909
	Sph	117.8648	177.9010	110.4694	117.8649	177.8975	110.4694	117.8649	177.8975	110.4694

Table 3. Mortality spatio-temporal kriging age-specific root-mean square error

Mortality age-specific group		Mortality root-mean square error
Infant	($x < 1$ years old)	3.7E-12
Pre-school	($1 \leq x < 4$ years old)	2.7E-15
School	($4 \leq x < 14$ years old)	2.1E-16
Productive	($14 \leq x < 64$ years old)	4.9E-11
Post-productive	($x \geq 64$ years old)	1.8E-09

2 Supplementary figures

Sample variogram and fitted covariance models

The winner permutations taken by one, two or three variograms (Exponential, Gaussian and/or Spherical) for independent spatio, temporal and joint spatio-temporal models according to the covariance structure (metric, separable, productSum, sumMetric and simpleSumMetric) are presented for post-productive, productive, school, pre-school and infant mortalities in Supplementary Figures 2-5 respectively.

Fig 1. Sample variogram and fitted covariance models for post-productive mortality.

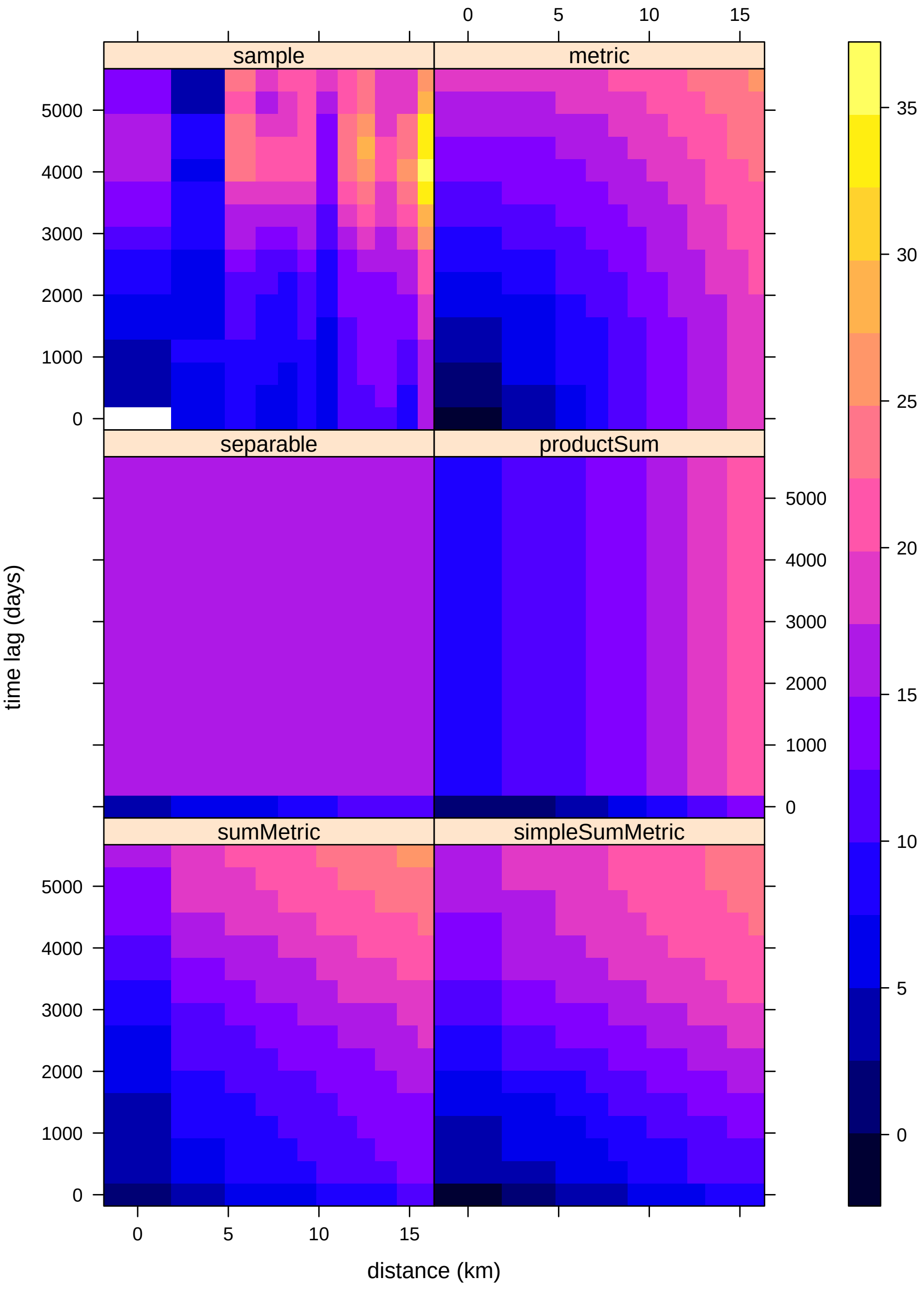

Fig 2. Sample variogram and fitted covariance models for productive mortality.

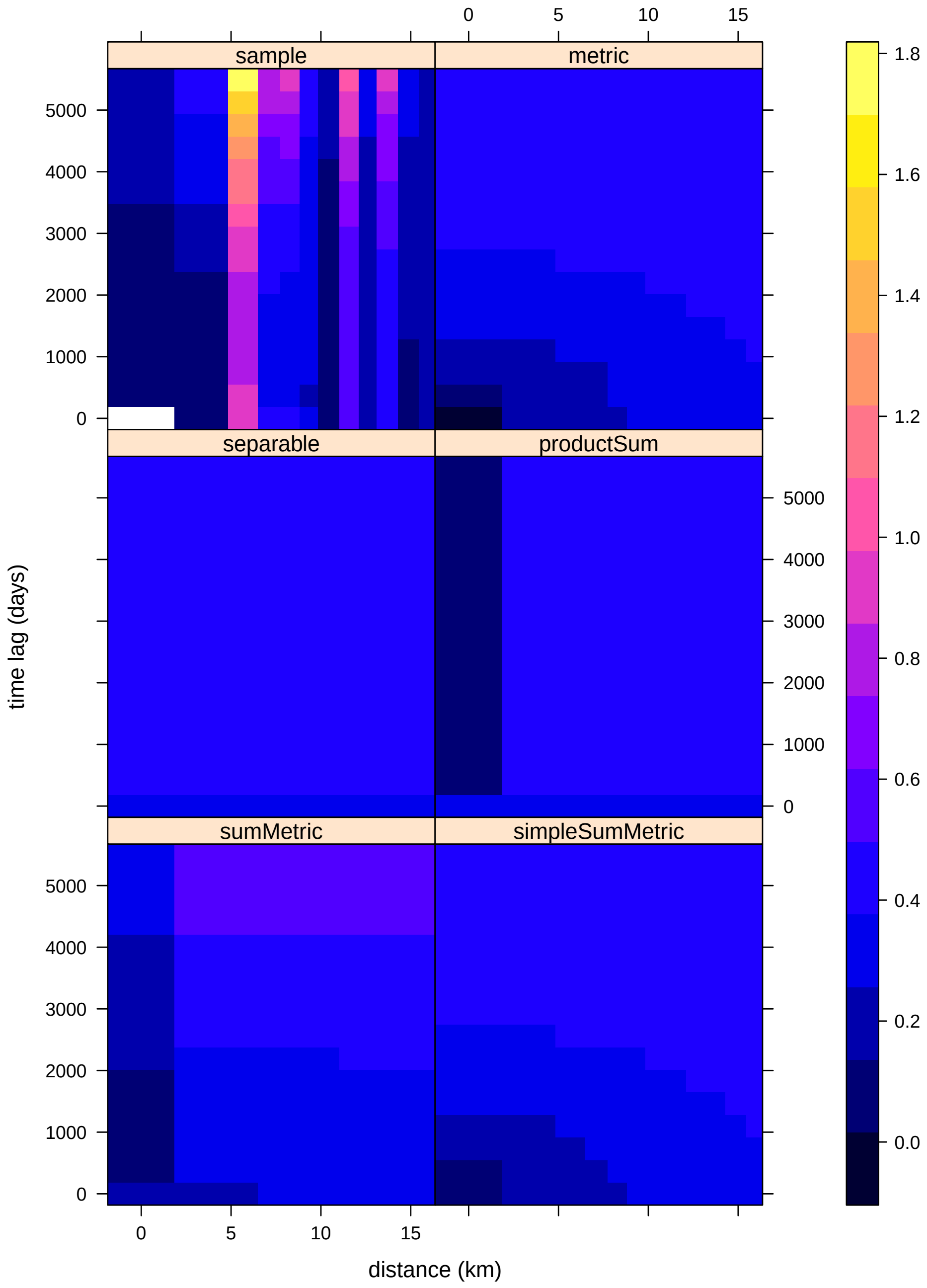

Fig 3. Sample variogram and fitted covariance models for school mortality.

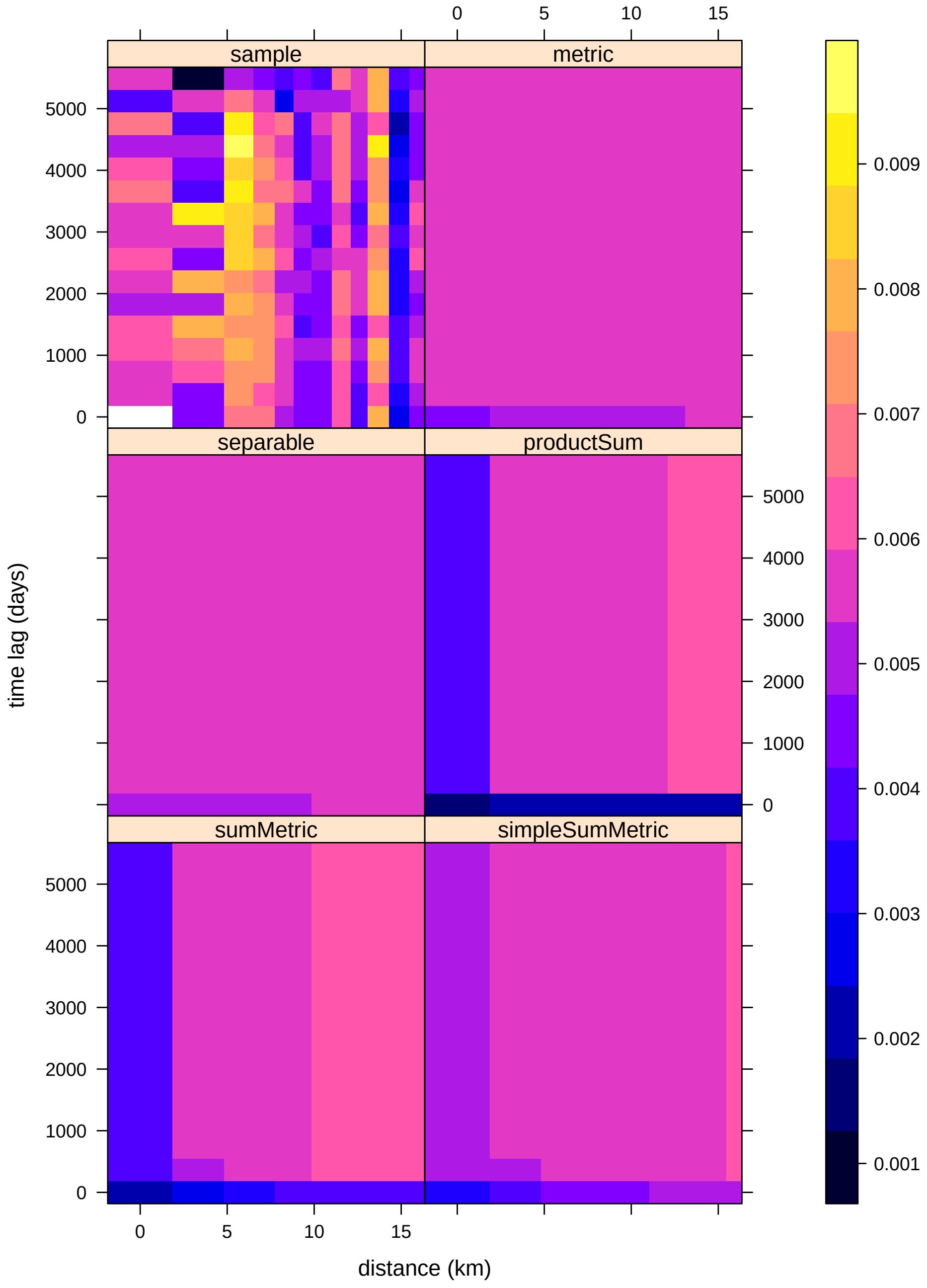

Fig 4. Sample variogram and fitted covariance models for pre-school mortality.

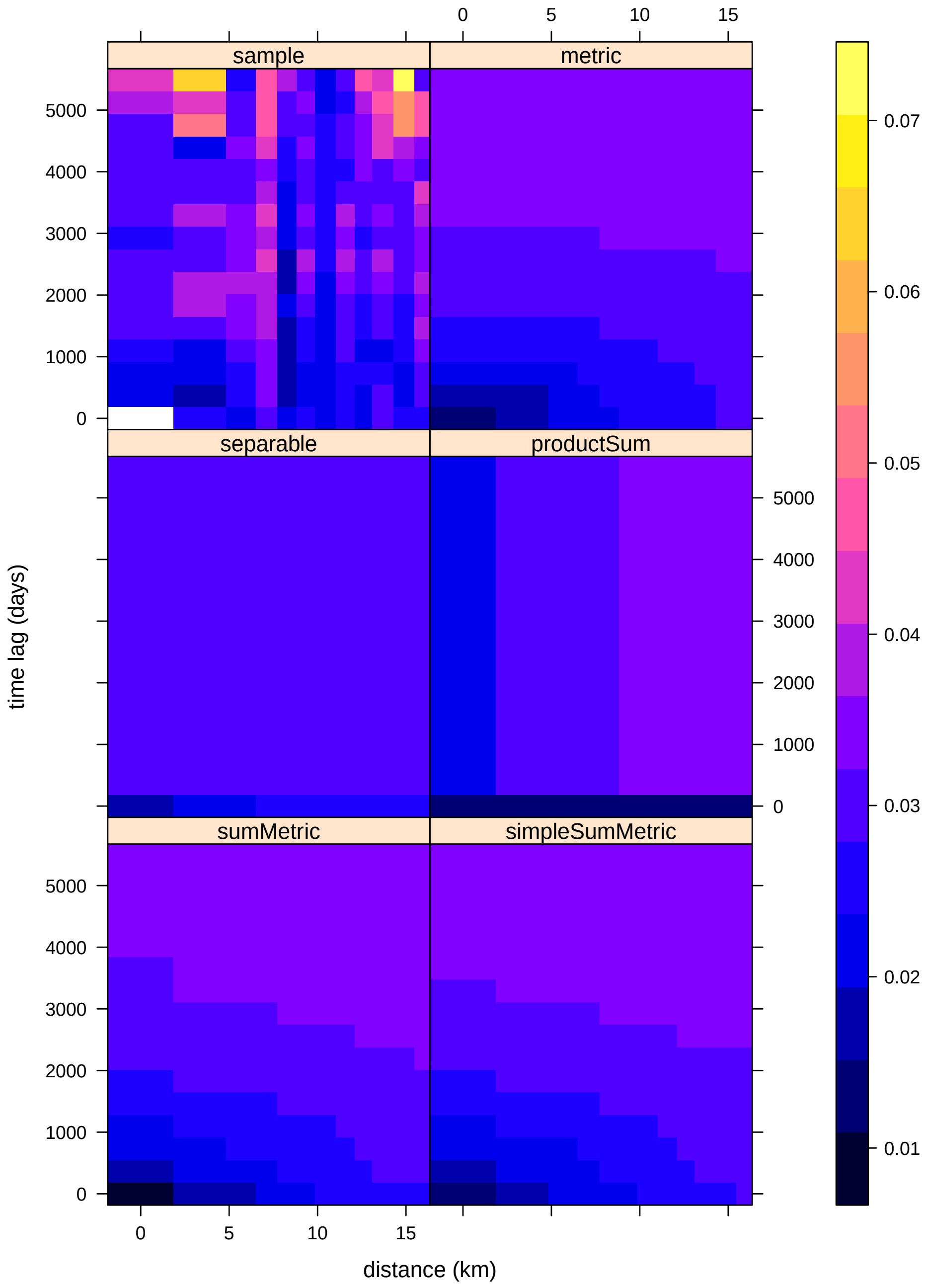

Fig 5. Sample variogram and fitted covariance models for infant mortality.

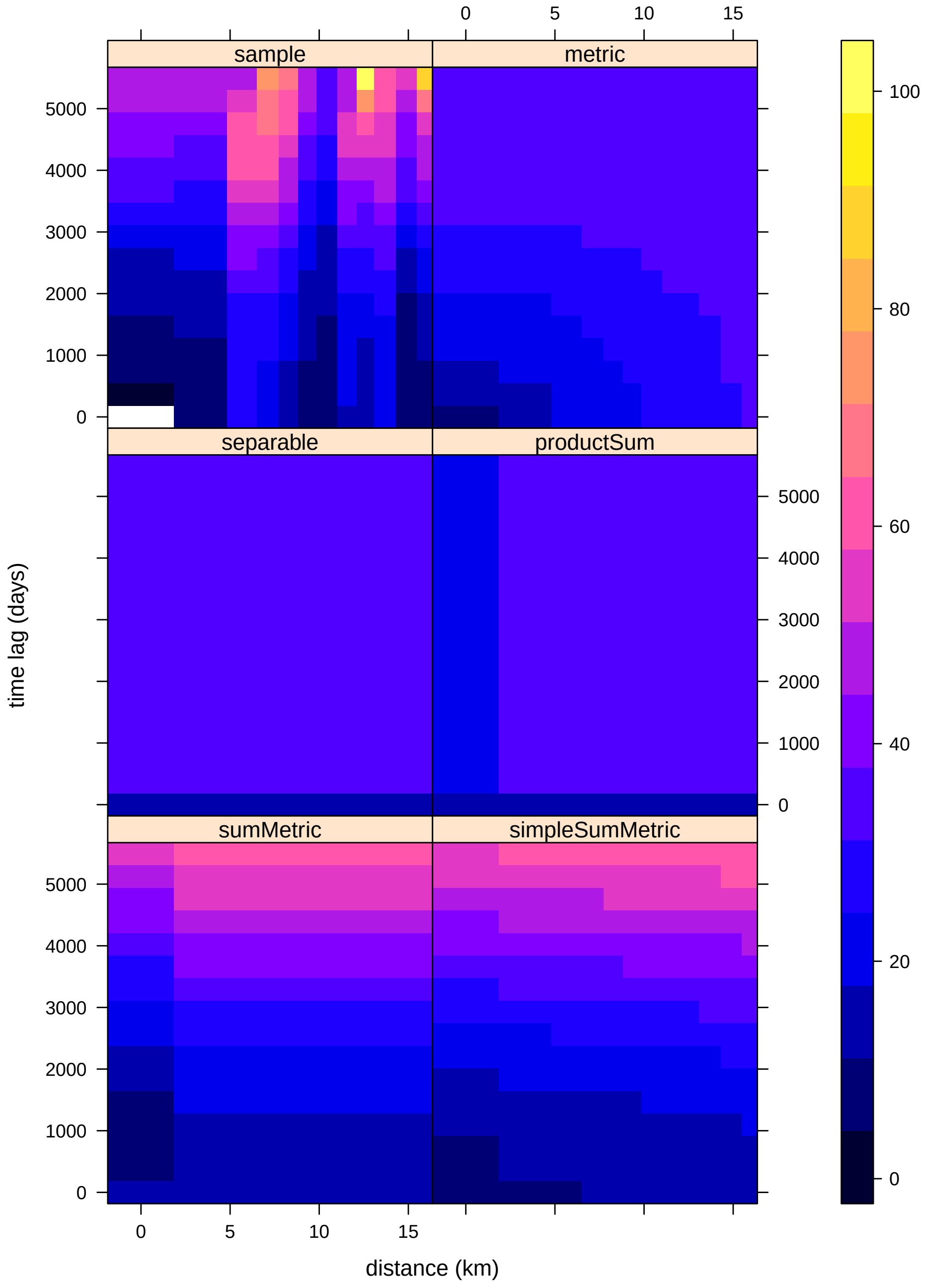

Supplement: S1 File — (PDF) [file pone.0244384.s001.pdf]
