# Supplementary material for: Metropolitan age-specific mortality trends at borough and neighborhood level: The case of Mexico City
Source: PLoS One. 2021 Jan 19;16(1):e0244384. doi: 10.1371/journal.pone.0244384 (PMC7815139; doi:10.1371/journal.pone.0244384)

S2 - Metropolitan age-specific mortality trends at borough and neighbourhood level: The case of Mexico City

Karol Baca-López[1,2¶], Cristóbal Fresno[3¶], Jesús Espinal-Enríquez[2], Miriam V. Flores-Merino[4], Miguel A. Camacho-López[1], Enrique Hernández-Lemus[2,5*]

[1] School of Medicine, Autonomous University of the State of Mexico, Toluca, State of Mexico, Mexico
[2] Computational Genomics Department, National Institute of Genomic Medicine, Mexico City, Mexico
[3] Technology Development Department, National Institute of Genomic Medicine, Mexico City, Mexico
[4] School of Chemistry, Autonomous University of the State of Mexico, Toluca, State of Mexico, Mexico
[5] Centro de Ciencias de la Complejidad, Universidad Nacional Autonoma de Mexico, Mexico City, Mexico

* Corresponding author E-mail: ehernandez@inmegen.gob.mx (EHL) ¶These authors contributed equally to this work.

1 Supplementary figures

Three levels of granularity for infant, pre-school and post-productive mortality rates in Mexico City.

Fig 1. Three levels of granularity for Infant mortality rate in Mexico City.

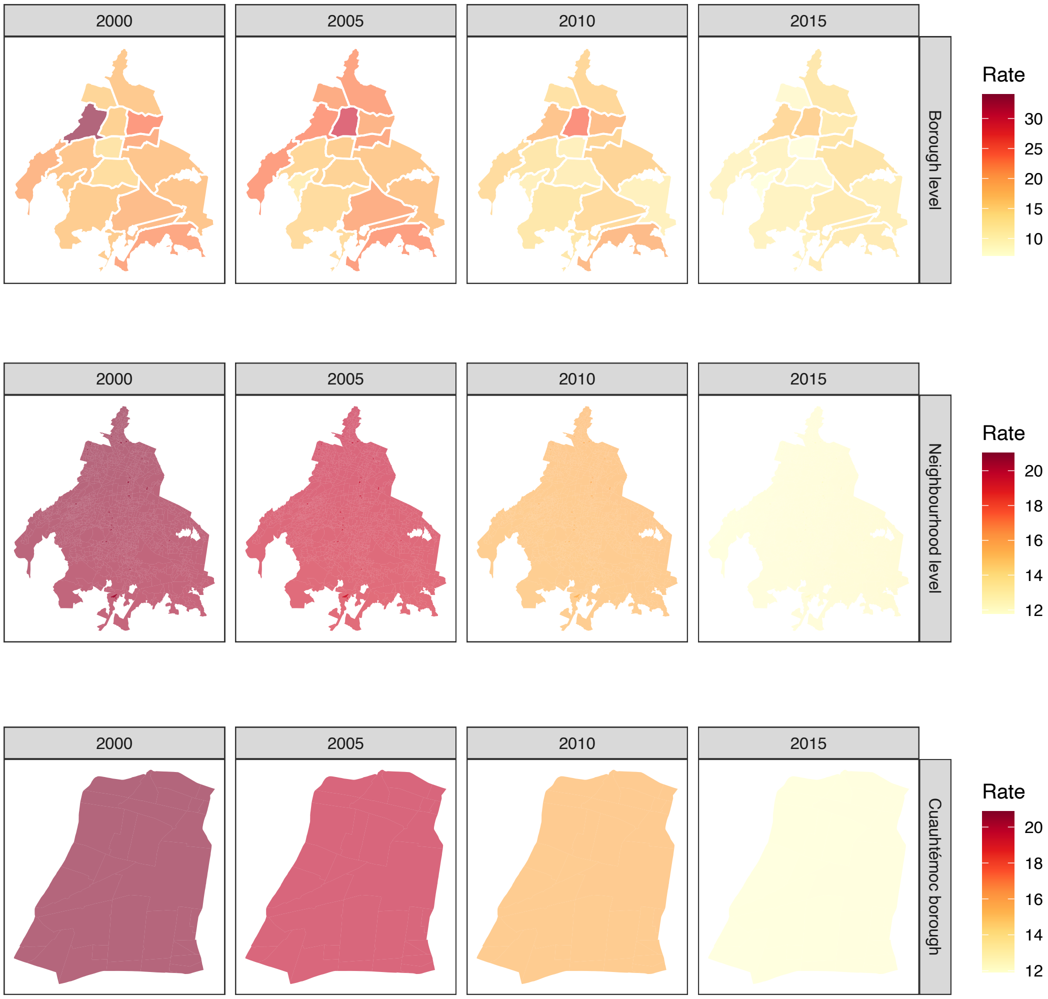

Fig 2. Three levels of granularity for Pre-school mortality rate in Mexico City.

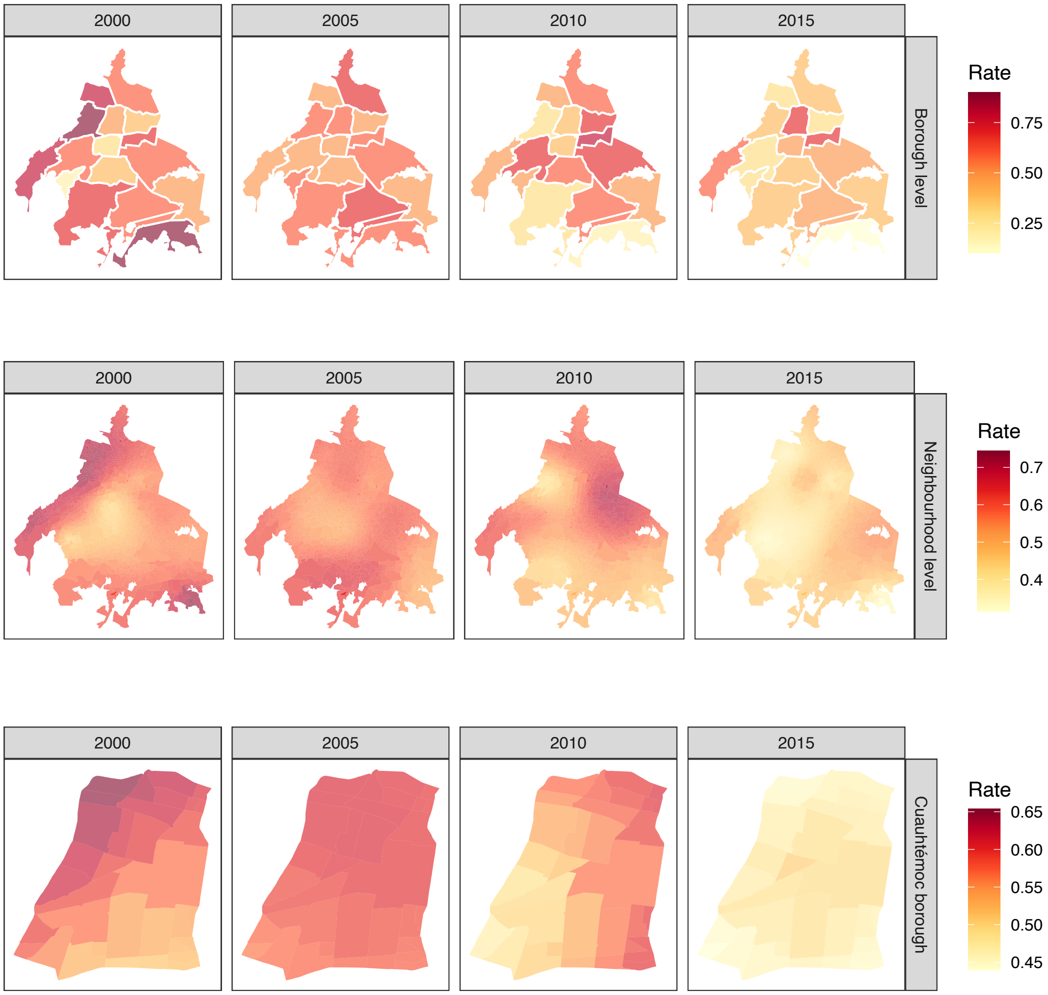

Fig 3. Three levels of granularity for Post-productive mortality rate in Mexico City.

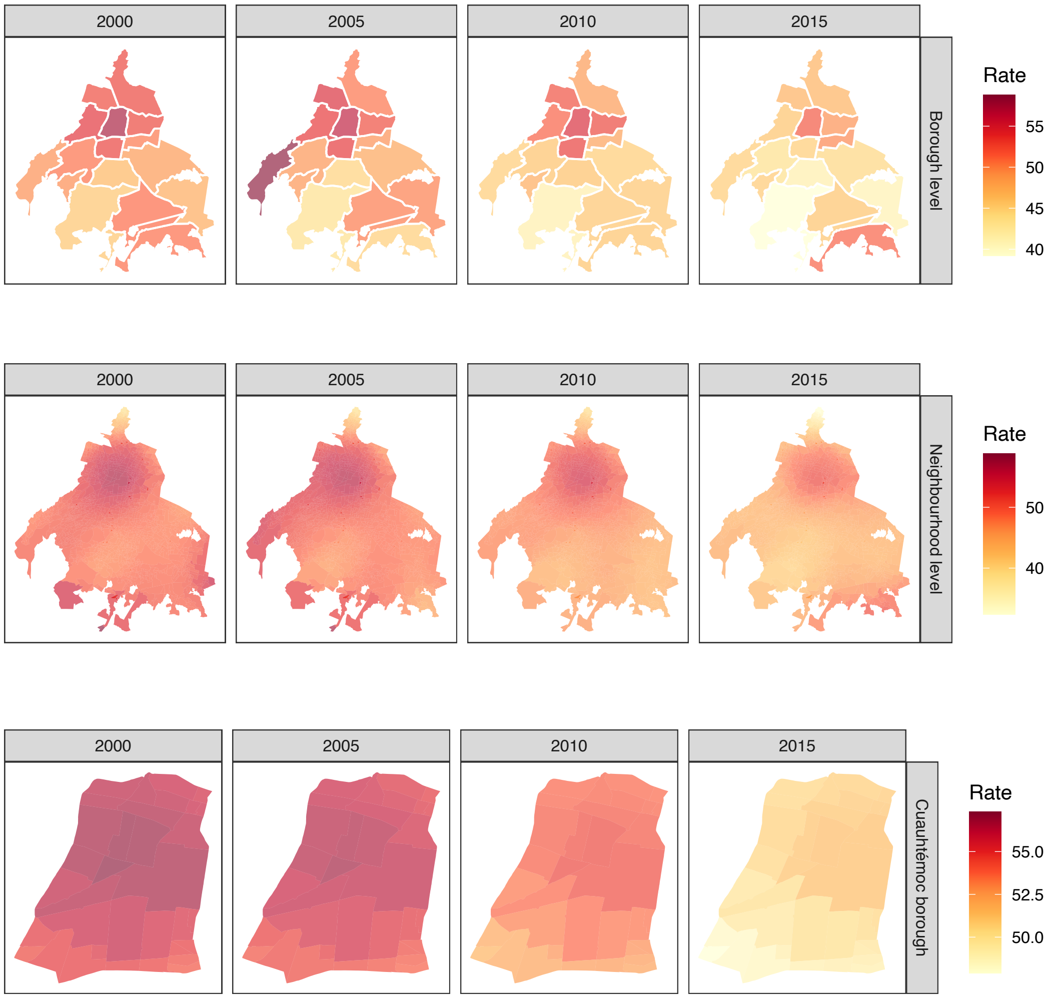

Supplement: S2 File — (PDF) [file pone.0244384.s002.pdf]
