# Supplementary material for: Metropolitan age-specific mortality trends at borough and neighborhood level: The case of Mexico City
Source: PLoS One. 2021 Jan 19;16(1):e0244384. doi: 10.1371/journal.pone.0244384 (PMC7815139; doi:10.1371/journal.pone.0244384)
Supplement: Attachment — Submitted filename: response2reviewers.pdf [file pone.0244384.s003.pdf]

Metropolitan age-specific mortality trends at borough and neighborhood level: The case of Mexico City

Karol Baca-López[1,2¶], Cristóbal Fresno[3¶], Jesús Espinal-Enríquez[2], Miriam V. Flores-Merino[4], Miguel A. Camacho-López[1], Enrique Hernández-Lemus[2,5*]

[1] School of Medicine, Autonomous University of the State of Mexico, Toluca, State of Mexico, Mexico
[2] Computational Genomics Department, National Institute of Genomic Medicine, Mexico City, Mexico
[3] Technology Development Department, National Institute of Genomic Medicine, Mexico City, Mexico
[4] School of Chemistry, Autonomous University of the State of Mexico, Toluca, State of Mexico, Mexico
[5] Centro de Ciencias de la Complejidad, Universidad Nacional Autonoma de Mexico, Mexico City, Mexico

* Corresponding author
E-mail: ehernandez@inmegen.gob.mx (EHL)
¶These authors contributed equally to this work.

Response to reviewers

PLOS ONE
Academic Editor
Dear Wenjia Zhang,

On behalf of the authors, I am glad to inform that all the comments/suggestions for article PONE-D-20-10411 have been properly addressed. We are sure that by following the reviewer's advice we have improved the quality of our manuscript and its readability. Please find the corresponding answers below.

Kind regards,

Prof. Enrique Hernández-Lemus
Corresponding author

Editor's comments

1. Focuses on the question of 'downscale' at the neighborhood level raised by Reviewer 2 2. finding clarification

Answer: The authors appreciate the editor's suggestion, which has been addressed in the revised version of the manuscript.

2. Language editing

Answer: In order to tackle this point, the manuscript has been revised by a native speaker.

3. The comparison of results at borough and neighborhood levels needs to address the modifiable areal unit problem (MAUP) and more about why these two scales.

Answer: The MAUP may, as the Editor points out, imply that a bias is generated affecting statistical hypothesis testing due to the combination of two or more spatial scales. This is so, since data aggregation can be dependent on the choice of modifiable areal unit (MAU) used as a primary source in our analysis. MAUP will in this case constitute on statistical biases that may lead to a form of ecological fallacy. In the particular case we have been studying here, since Kriging analysis is a form of regression, there are some ways to solve the MAUP. We decided to follow a mixed approach by incorporating several of these as follows:

(a) One of the alternatives is *to correct the variance-covariance matrix using samples from individual-level data*

Indeed, to this first end, we have tackled the MAUP in a hierarchical manner:

- Spatio-temporal kriging itself was modeled first, selecting the best combination of spatio-temporal variance-covariance structure (metric, separable, productSum, sumMetric and simpleSumMetric) with the appropriate single, double or triple variogram combinations (Exponential, Gaussian and Spherical), to minimize the weighted mean squared error (see S1 File Table 2 and 3). Once the complete variance-covariance has been fixed, the neighborhood spatio-temporal grid was used to obtain the kriged values.
- Over the original data, at borough level, a linear mixed-effect model using the definitions of Eq (3- 6). In these definitions, as in Kwok et al. 2008 [1], we did include a first-order autoregression (AR(1)) structure. In addition, data heteroscedasticity was modeled as $var(\varepsilon_{ijk}) = \sigma^2 g^2(\alpha_i)$, i.e., a multiplicative effect of the residual variance σ^2 times the variance error function $g(.)$ using a `varIdent` definition for the different age-specific mortality groups α_i, as described in Material an Methods section.
- Over the kriged data, at the neighborhood level, also the model of Eq (3-6) was fitted to cope with the data, i.e. considering a different MAU to meet our needs.

(b) A second way to correct for the MAUP is to *focus on local spatial regression rather than global regression*

Here, we used a global regression for the kriging process, but, considering the best variance-covariance structure possible for our data, letting the model to adjust its contribution instead of using a fixed local spatio-temporal regression. The RMSE for each mortality age-specific group is presented in S1 File Table 3 and here in Table 2.

(c) A third strategy to correct the MAUP bias is by resorting to *design areal units to maximize a particular statistical result.* In this manuscript, we first:

   - Maximized the model likelihood while minimizing the weighted mean squared error when selecting the best spatio-temporal variance-covariance structure.
   - Maximized the linear mixed-effect model likelihood for both borough and/or neighborhood data level description.

(d) Others have suggested *developing statistics that change across scales in a predictable way*, perhaps using fractal dimension as a scale-independent measure of spatial relationships.

   The authors did not include statistics that change across scales in a predictable way. However, in our model the change of scale was made instead using independent linear mixed-effect models.

(e) Others have suggested *Bayesian hierarchical models as a general methodology for combining aggregated and individual-level data* for ecological inference.

   This was not the strategy used in this manuscript. This is so due to the incompleteness of our initial data that will have a severe impact in the priors severely diminishing their unbiasing power.

(f) A final way may be *using simulated data.* Data simulation is necessary to have control over various properties of individual-level data. *Simulation studies have demonstrated that the spatial support of variables can affect the magnitude of ecological bias caused by spatial data aggregation.*

   No simulated data was included in the scope of this work to control individual-level data.

Hence, out of the six main strategies to correct for MAUP biases, in this work we have resorted to using a mixed approach including three of these.

4. For the mixed-effect linear model, while this type of models have been widely used in varying related fields, more literature reviews about them are needed to make your model analytically sound, including those similar models using different names, like multilevel modeling of longitudinal data, longitudinal multilevel model, and longitudinal HLM. You may refer to some literature below:

   - Raudenbush, S. W., and A. S. Bryk. A Hierarchical Model for Studying School Effects. Sociology of Education, Vol. 59, No. 1, 1986, pp. 1–7.
   - Kwok, O.M., Underhill, A.T., Berry, J.W., Luo, W., Elliott, T.R. and Yoon, M., 2008. Analyzing longitudinal data with multilevel models: an example with individuals living with lower extremity intra-articular fractures. Rehabilitation psychology, 53(3), p.370.
   - Zhang, W. and Zhang, M., 2015. Short-and long-term effects of land use on reducing personal vehicle miles of travel: Longitudinal multilevel analysis in Austin, Texas. Transportation Research Record, 2500(1), pp.102-109.

Answer: After a thorough review of the literature proposed by the reviewer, the authors arrived at the following conclusions:

- As stated by the reviewer, according to the field at hand, the used models have proper names in order to better reflect certain features of the data or the data modeling process itself. Such is the case of including particular keywords within the proper name of the model, e.g., longitudinal to refer to

the data nature in a time series or multilevel or hierarchical to refer to the modeling process. Proper names examples include multilevel modeling for longitudinal data, longitudinal multilevel model, and longitudinal hierarchical linear model.

- In any of the used models, the authors are trying to model the data, taking advantage of the variance-covariance structure of the data:
  - Raudenbush & Bryk 1986 [2] took advantage of observed unit (individuals) when considering between and within school effects. Hence, their proposal can bee seen as a hierarchical or multilevel approach, where first, the within-group model is estimated by a separate regression equation for each school (referenced as Eq 1 in the original work). Then, the between-group model uses the regression coefficients as dependent output and try to model the within-school structural relationships (Eq 2 in [2]). Finally, the Raudenbush & Bryk can be coupled into a single equation by substitution of Eq 2 into Eq 1. The resulting equation allows to model the error term with many degrees of freedom associated with the studied variables of interest. However, the model estimation itself is not explained in detail.
  - Kwok et al. 2008 [1] are more concerned about modeling variable time data points (not necessary at fixed intervals), unlike repeated-measurements Analysis of the Variance (ANOVA) or ANOVA polynomial trend analysis. They propose to model the variance structure by means of linear growth models using multilevel models. They start with a simple random intercept model and simple linear growth model, to show how the variance-covariance structure can tackle within-class correlation. Then, they move to models with time-invariant covariate and pseudo-R^2 statistic to evaluate the model's effectiveness. Finally, the present how to model covariance structure for the within-individual random errors were, the proximal autocorrelation in longitudinal data, was addressed using a first-order autoregression (AR(1)) structure. In this manuscript, the authors fully detailed how the estimations are made by means of using SPSS (MIXED) and SAS (PROC MIXED) procedures.
  - Zhang & Zhang 2015 [3] use a Longitudinal Multilevel Model (LMM) at two data level description (household–individual and traffic analysis zones) using Raudenbush & Bryk approach. The authors do mention that STATA software was used but, there is no detailed description of the procedures used to estimate the model's coefficients.
- If we turn back our attention to Kwok et al. 2008 work [1], the manuscript includes a detailed description of SPSS (MIXED) and SAS (PROC MIXED) procedures used. Looking deeper into both software manuals for these specific procedures over the following links `https://www.spss.ch/upload/1126184451_Linear%20Mixed%20Effects%20Modeling%20in%20SPSS.pdf` and `https://support.sas.com/documentation/onlinedoc/stat/141/mixed.pdf` we have found that:
  - The SPSS manual is called itself "Linear Mixed-Effects Modeling in SPSS: An Introduction to the MIXED Procedure". Hence, linear growth models are essentially modeled by linear mixed-effects models.
  - The SAS user's guide for the mixed procedure, also explains how to fit a variety of mixed linear models.

- In essence a linear mixed-effect model generalizes the standard linear model

$$y = X\beta + Z\gamma + \varepsilon \quad (1)$$

where, y, represents a vector of observed data; β and γ are unknown vectors of fixed and random effects parameters, with known design matrix X and Z respectively; and, ε is the residual error vector. If γ and ε are assumed to be Gaussian random variables that are uncorrelated and have expectation 0 and variance G and R, respectively. The variance of y can be modeled by (2):

$$V = ZGZ' + R \quad (2)$$

Hence, the standard linear model implies that $Z = 0$ and $R = \sigma^2 I$. However, different configurations of G, Z and R will define the flexible variance-covariance structure of y. In our manuscript, as in Kwok et al. 2008 [1], we did include a first-order autoregression (AR(1)) structure. In addition, data heteroscedasticity was modeled as $var(\varepsilon_{ijk}) = \sigma^2 g^2(\alpha_i)$, i.e., a multiplicative effect of the residual variance σ^2 times the variance error function $g(.)$ using a `varIdent` definition for the different age-specific mortality groups α_i, as described in Materials and Methods.

- Recent models by Anaya & Al-Delaimy 2017 [4] explicitly mention to use a "*mixed effect linear model with random effect estimation and repeated measurements*" in their work. Additionally, the hierarchical linear model in [5] and the multilevel modeling of longitudinal data in [6] were fitted using SPSS (MIXED) and SAS (PROC MIXED) procedures respectively. On the other hand, Green et al. 2019 [7] used linear mixed-effect models with R software [8] by means of `lme4` package, to fit multilevel models with random intercepts for schools and random intercepts and random slopes for time at the individual level since students are nested within waves.

Journal Requirements

1. Please ensure that your manuscript meets PLOS ONE's style requirements, including those for file naming. The PLOS ONE style templates can be found at:
   - `https://journals.plos.org/plosone/s/file?id=wjVg/PLOSOne_formatting_sample_main_body.pdf`
   - `https://journals.plos.org/plosone/s/file?id=ba62/PLOSOne_formatting_sample_title_authors_affiliations.pdf`

Answer: The authors have checked Plos One's journal requirements, including those for file naming, in the revised version of the manuscript.

2. We note that Figures 1, 2, 4 and 6 in your submission contain map images which may be copyrighted. All PLOS content is published under the Creative Commons Attribution License (CC BY 4.0), which means that the manuscript, images, and Supporting Information files will be freely available online, and any third party is permitted to access, download, copy, distribute, and use these materials in any way, even commercially, with proper attribution. For these reasons, we cannot publish previously copyrighted maps or satellite images created using proprietary data, such as Google software (Google Maps, Street View, and Earth). For more information, see our copyright guidelines:
   - `http://journals.plos.org/plosone/s/licenses-and-copyright.`

We require you to either (1) present written permission from the copyright holder to publish these figures specifically under the CC BY 4.0 license, or (2) remove the figures from your submission:

(a) You may seek permission from the original copyright holder of Figures 1, 2, 4 and 6 to publish the content specifically under the CC BY 4.0 license.

We recommend that you contact the original copyright holder with the Content Permission Form (`http://journals.plos.org/plosone/s/file?id=7c09/content-permission-form.pdf`) and the following text:

> *"I request permission for the open-access journal PLOS ONE to publish XXX under the Creative Commons Attribution License (CCAL) CC BY 4.0 (http://creativecommons.org/licenses/by/4.0/). Please be aware that this license allows unrestricted use and distribution, even commercially, by third parties. Please reply and provide explicit written permission to publish XXX under a CC BY license and complete the attached form."*

Please upload the completed Content Permission Form or other proof of granted permissions as an "Other" file with your submission.

In the figure caption of the copyrighted figure, please include the following text: "Reprinted from [ref] under a CC BY license, with permission from [name of publisher], original copyright [original copyright year]."

(b) If you are unable to obtain permission from the original copyright holder to publish these figures under the CC BY 4.0 license or if the copyright holder's requirements are incompatible with the CC BY 4.0 license, please either i) remove the figure or ii) supply a replacement figure that complies with the CC BY 4.0 license. Please check copyright information on all replacement figures and update the figure caption with source information. If applicable, please specify in the figure caption text when a figure is similar but not identical to the original image and is therefore for illustrative purposes only.

The following resources for replacing copyrighted map figures may be helpful:

- USGS National Map Viewer (public domain): `http://viewer.nationalmap.gov/viewer/`
- The Gateway to Astronaut Photography of Earth (public domain): `http://eol.jsc.nasa.gov/sseop/clickmap/`
- Maps at the CIA (public domain): `https://www.cia.gov/library/publications/the-world-factbook/index.html` and `https://www.cia.gov/library/publications/cia-maps-publications/index.html`
- NASA Earth Observatory (public domain): `http://earthobservatory.nasa.gov/`
- Landsat: `http://landsat.visibleearth.nasa.gov/`
- USGS EROS (Earth Resources Observatory and Science (EROS) Center) (public domain): `http://eros.usgs.gov/`
- Natural Earth (public domain): `http://www.naturalearthdata.com/`

Answer: As regards this journal's requirement, the authors understand and respect all forms of copyright (data, images, etc.). In this concern the authors did use the freely available map data from The National System of Statistical, Geographical and Informatics (INEGI, Instituto Nacional de Estadística, Geografía e Informática, México). In this concern at INEGI's webpage the following statements apply.

About us `http://en.www.inegi.org.mx/inegi/quienes_somos.html`
: We are an autonomous public body responsible for regulating and coordinating the National System of Statistical, Geographical and Informatics, as well as for collecting and disseminating information about Mexico in terms of territory, resources, population and economy, which makes it possible to know the characteristics of our country, and help in the decision making process.

Dissemination of information
: `http://en.www.inegi.org.mx/inegi/contenido/difusion.html`
The INEGI offers statistical and geographical information that can be consulted and downloaded, it has a great thematic diversity and different levels of aggregation, and it is completely free of charge. To provide access to information, it offers the following customer services to the public ...

Particularly, the following piece of information was obtained as primary data source:

(a) Geostatistic framework, December 2018 (Marco Geoestadístico, Diciembre 2018). Shape files for Mexico's country mini-map and Mexico City (complete, borough and neighborhood level).

- The data can be downloaded at `http://en.www.inegi.org.mx/app/biblioteca/ficha.html?upc=889463674658` where the user can select the complete geostatistic framework using the following link `http://internet.contenidos.inegi.org.mx/contenidos/productos/prod_serv/contenidos/espanol/bvinegi/productos/geografia/marcogeo/889463674658_s.zip`
- Once downloaded and unziped the complete 889463674658_s.zip file, inside the file called Marco_Geoestadistico_Integrado_diciembre_2018.zip there is the file metadatos/mg_int_dic18_31.txt which contains the specific use for these files:
  - For this manuscript, the authors fall into last sentence of section 1.15, assumption 1 (internal):

    "*This information is intended for institutions in the public and private sectors and the general public; Thus, decision-making is supported in government programs and social support, as well as research projects on the subject.*" (translated text)

    For a quick reference below is the complete original text for section 1.15, assumptions 1 (internal):

    "*1.15 Uso específico:*
    USO 1 (INTERNO): las direcciones generales de Estadísticas Sociodemográficas y de Estadísticas Económicas del INEGI emplean el Marco Geoestadístico para la realización de los diferentes tipos de censos y encuestas. Utilizan los catálogos y la cartografía geoestadística -impresa y digital- primero como insumos para la planeación y organización del operativo, posteriormente para el levantamiento y control de cobertura territorial y finalmente para la organización, presentación y divulgación de resultados. Se hace con el apoyo de herramientas informáticas que facilitan la obtención, captación y almacenamiento de los datos. Se generan así estadísticas sociales y económicas, con referencia geográfica, basadas en una determinada unidad de observación:

vivienda, establecimiento económico. Dicha información se destina a instituciones de los sectores público y privado y al público en general; se apoya así la toma de decisiones en programas de gobierno y apoyos sociales, así como proyectos de investigación en la materia.

– For this manuscript, also applies section 8.2 Restrictions of use: None. For a quick reference the original text is the following: *"8.2 Restricciones de uso: 8. Ninguna."*

Data manipulation for figures included in the article

The authors declare that all maps were based on INEGI's shape files data and manipulated to report results based on our mortality data modeling using R software [8] and the following libraries: `sf` [9], `rgeos` [10], `raster` [11], `geosphere` [12], `spacetime` [13], `sp` [14], `rgdal` [15], `ggplot2` [16], `cowplot` [17], `gridExtra` [18] and `ggspatial` [19]. Hence, no copyrighted materials were used in this regard.

The authors agree that no copyright issues has been inflicted, and also agree to use Creative Commons Attribution License (CC BY 4.0), according to Plos One regulations, upon their figures and manuscript are published.

3. We note that you have included the phrase "data not shown" in your manuscript. Unfortunately, this does not meet our data sharing requirements. PLOS does not permit references to inaccessible data. We require that authors provide all relevant data within the paper, Supporting Information files, or in an acceptable, public repository. Please add a citation to support this phrase or upload the data that corresponds with these findings to a stable repository (such as Figshare or Dryad) and provide and URLs, DOIs, or accession numbers that may be used to access these data. Or, if the data are not a core part of the research being presented in your study, we ask that you remove the phrase that refers to these data.

Answer: The authors agree to comply with PLOS' data sharing requirements. Hence, the manuscript was modified accordingly in order to remove the phrase that refers to these data, as the data are not a core part of the research being presented in your study, as suggested by the Journal's requirements at the two places where data not shown was referenced:

Original version 1 In addition, neighborhood centroids were obtained by gCentroid or gPointOnSurface for convex or concave polygons respectively (data not shown).

Revised version 1 In addition, neighborhood centroids were obtained by `gCentroid` or `gPointOnSurface` for convex or concave polygons respectively.

Original version 2 Although apparently imperceptible, there are distinguishable differences in mortality values among Cuauhtemoc neighborhoods. Similar difference are obtained for the rest of the boroughs (data not shown).

Revised version 2 Although apparently imperceptible, there are distinguishable differences in mortality values among Cuauhtemoc neighborhoods and a similar behavior for the rest of the boroughs is found, as seen in the borough level description Fig 4 B.

4. While revising your submission, please upload your figure files to the Preflight Analysis and Conversion Engine (PACE) digital diagnostic tool, `https://pacev2.apexcovantage.com/`. PACE helps ensure that figures meet

PLOS requirements. To use PACE, you must first register as a user. Registration is free. Then, login and navigate to the UPLOAD tab, where you will find detailed instructions on how to use the tool. If you encounter any issues or have any questions when using PACE, please email PLOS at figures@plos.org. Please note that Supporting Information files do not need this step.

Answer: The revised versions of the figures have been checked for PACE compliance.

5. PLOS authors have the option to publish the peer review history of their article (what does this mean?). If published, this will include your full peer review and any attached files. If you choose "no", your identity will remain anonymous but your review may still be made public.

Answer: The authors have decided to include the peer review history of our article.

Reviewer #1

1. This paper would benefit from English editing.

Answer: In order to tackle this point, the manuscript has been revised by a native speaker.

2. Line 19. Mortality can be explained by individual level characteristics and population level variables. Is this what the authors mean, or do the authors want to emphasize the difference between individual level mortality (e.g. frailty) and population level mortality? I suggest rephrasing this sentence to make clearer what the authors want to express.

Answer: The authors have rephrased the original idea in order to make a better point of view on the subject.

Original version On the other hand, mortality as well as other health outcomes, can equally be described at different scales, for example, individually and collectively.

Revised version In addition, age-specific mortality can be influenced by social determinants at the individual level and at national or state level.

3. Line 34. I don't consider all-cause and cause-specific opposite approaches since the sum of cause-specific gives all-cause mortality.

Answer: The authors have rephrased the original idea to comply with the reviewer's suggestion.

Original version Regarding the description of spatial mortality trends, there exist basically two main opposite approaches: i) All-cause or ii) Cause-specific mortality studies.

Revised version Regarding the description of spatial mortality trends, there exist basically two main approaches: i) All-cause or ii) Cause-specific mortality studies.

4. The 4th (starting at line 51) and 5th paragraph seems out of place and cuts the flow of the introduction. I suggest changing this paragraph to the methodology section since is about the data and different models used.

Answer: The paragraph has been now included in the materials and methods section.

5. There are other recent studies that also look at state-level mortality with a focus on violent deaths and other diseases that could be referenced to on cause-specific trends in Mexico City:

- Gómez-Dantés, H., Fullman, N., Lamadrid-Figueroa, H., Cahuana-Hurtado, L., Darney, B., Avila-Burgos, L., ... & Aburto-Soto, T. (2016). Dissonant health transition in the states of Mexico, 1990–2013: a systematic analysis for the Global Burden of Disease Study 2013. The Lancet, 388(10058), 2386-2402. [gomez2016]
- Aburto, J. M., Beltrán-Sánchez, H., García-Guerrero, V. M., & Canudas-Romo, V. (2016). Homicides in Mexico reversed life expectancy gains for men and slowed them for women, 2000–10. Health Affairs, 35(1), 88-95. [aburto2016]
- Aburto, J. M., & Beltrán-Sánchez, H. (2019). Upsurge of homicides and its impact on life expectancy and life span inequality in Mexico, 2005–2015. American journal of public health, 109(3), 483-489. [aburto2019]

Answer: The following paragraph was added to the introduction to give some context about risk factors for health outcomes and mortality in Mexico (nationwide) and Mexico City. The suggested references were cited within the manuscript.

> "*To set some context of mortality causes and trends in Mexico City, in a recent study, Aburto et al. (2018) analyzed lifespan and preventable mortality for Mexico City and the other 31 states. They described changes for age groups and particularly reported an increase in Diabetes and heart diseased-related mortality rates. Moreover, beyond diseased related mortality causes, it is well know that an important number of deaths in Mexico City corresponds to homicides. Aburto et al. conducted different studies for the periods 2000-2010 and 2005-2015 regarding the impact of homicides in life expectancy and life-span inequality. The authors reported an increase in homicide mortality that surpassed positive outcomes from health care reforms oriented to promote life expectancy at the national level. Regarding health outcomes, Gómez-Dantés, et al. (2016) report the leading risk factors for children and adults. Supporting the fact that public health reforms and interventions should vary according to the specific risk factor for each age group. To give some examples, diarrhoeal diseases, under nutrition and poor sanitation for children and chronic diseases and violence for adults.*"

6. Fig 4. One of the contributions of the paper is looking at more fine age groups than just looking at the all-mortality. In Fig 4, caution should be made if the rate was calculated by only dividing deaths/population*1000. Without standardizing by age-group, comparisons over time and between boroughs are inadequate. For example, the increase in the rate could be a result of the aging process and not that mortality is increasing per se. I suggest focusing on age-group mortality throughout the paper (without global) because of the mentioned reason.

Answer: In concordance and agreement with the reviewer's comment, the authors have removed global mortality throughout the manuscript (text and figures). Also, Fig 4-5 have been included to focus in two age-groups. The complementary age-groups have been included in S2 File as supplementary figures.

7. Fig 5. It would be good to explain more about the LSD groups and what they mean.

Answer: The reviewer's suggestion has been taken into consideration and the revised version of the manuscript includes more information about LSD groups and their interpretation.

8. Fig 6. The mortality rate shown in Fig 6 is nor consistent with Fig 5, e.g. a mortality rate of almost 8 for post productive group, and in Fig 5 is above 45. What are the explanations for this discrepancy?

Answer: The authors would like to give a proper explanation to discern the possible discrepancy that the reviewer point out. The mortality data has been modeled using a mixed-effect linear model using the definitions of Eq (3- 6) which are transcribed here:

$$\begin{aligned} y_{ijk} = \mu + \alpha_i + \beta_j + \gamma \times t_k + \delta \times t_k^2 + \zeta \times t_k^3 + \alpha_i \times \beta_j + \alpha \times \gamma \times t_k + \\ \alpha_i \times \delta \times t_k^2 + \alpha_i \times \zeta \times t_k^3 + \beta_j \times \gamma \times t_k + \beta_j \times \delta \times t_k^2 + \beta_j \times \zeta \times t_k^3 + \\ \alpha_i \times \beta_j \times \gamma \times t_k + \alpha_i \times \beta_j \times \delta \times t_k^2 + \\ \alpha_i \times \beta_j \times \zeta \times t_k^3 + \varepsilon_{ijk} \end{aligned} \quad (3)$$

$$\varepsilon_{ijk} = \lambda_{t_k} + \nu_{ijk} \quad (4)$$

$$\lambda_{t_k} = \phi_{\beta_j} \lambda_{t_{k-1}} + u_{t_k} \quad (5)$$

$$var(\epsilon_{ijk}) = \sigma^2 g^2(\alpha_i) \quad (6)$$

where, y_{ijk} is the mortality rate for the i-th age-specific mortality group (α_i), at the j-th borough or neighborhood level (β_j), for the k-th time (t_k); μ is the global mean; γ, δ, ζ are the corresponding third order time polynomial coefficients; in addition, the double and triple complete fixed effects model interactions and the error term ε_{ijk} were also specified. Indeed, the error term ε_{ijk} in Eq (3) is modeled in Eq (4) by a two-level model, to account for the lack of independent errors; where, λ_{t_k} denotes the unobservable time effect and ν_{ijk} is the idiosyncratic error term. The correlated errors were tackled using a first order auto-regressive model for time dependence as described by Eq (5), where ϕ_{β_j} is the corresponding coefficient and u_{t_k} the individual effect. Finally, the heteroscedasticity in Eq (6) was modeled as a multiplicative effect of the residual variance σ^2 times the variance error function $g(.)$ using a varIdent definition for the different age-specific mortality groups α_i [20]. The model Analysis of the Variance table, using Type III sum of squares to assess model's result are shown in Table 1.

Considering the aforementioned data modeling, the authors want to better describe the obtained model results. First, mortality data was independently fitted using the same framework defined in Eq (3- 6), where, according to the corresponding scenario the differences are:

Borough level: Here, the fixed effect term, β_j, stands for each of $j-th$ boroughs in Mexico City (16 in total). Hence, the observed mortality is, indeed, the original longitudinal data points freely available at the Secretariat of Health in Mexico City (Secretaría de Salud de la Ciudad de México: SEDESA) [21]. Actually, $y_{ijk} = y_{ijk}|_{\beta_j = Borough_j}$ given the fact that we are at borough level model.

Neighborhood level: Here, the fixed effect term, β_j, stands for each of $j-th$ neighborhood in a single borough of the Mexico City, e.g. $\beta_j|_{Borough=Cuauhtemoc}$ for the neighborhoods in Cuauhtemoc's borough. Unlike, the model at the Borough Level, this model uses as observed mortality, the spatio-temporal mortality krigged data according to the neighbor's centroid (latitude and longitude coordinates), thus, $y_{ijk} = y_{ijk}|_{\beta_j = Neighbourhood_j|_{Borough=Cuauhtemoc}}$

The results depicted in Table 1 showed for each of the models, the significant terms included in the final current model after removing not relevant terms, following parsimony principle. For example, there is no significant neighborhood

Table 1. Analysis of the Variance at borough and neighborhood level

Model Term	Borough level			Neighborhood level		
	Degrees of freedom	F-value	p-value	Degrees of freedom	F-value	p-value
μ	1	17634.6381	<0.0001	1	281297.3649	<0.0001
α_i	5	6981.1137	<0.0001	5	6929.1962	<0.0001
β_j	15	8.3045	<0.0001	33	6.5863	<0.0001
t	1	7.9808	0.0048	1	714.5003	<0.0001
t^2	1	34.1072	<0.0001	1	248.0783	<0.0001
t^3	1	33.6751	<0.0001	1	430.7367	<0.0001
$\alpha_i \times \beta_j$	75	20.0209	<0.0001	165	5.6587	<0.0001
$\beta_j \times t$	15	2.9735	0.0001	33	9.0428	<0.0001
$\beta_j \times t^2$	15	2.4779	0.0013	33	2.2043	<0.0001
$\beta_j \times t^3$	15	1.9233	0.0177			
$\alpha_i \times t$	5	4.172	0.0009	5	69.3982	<0.0001
$\alpha_i \times t^2$	5	7.2432	<0.0001	5	88.1181	<0.0001
$\alpha_i \times t^3$	5	7.4925	<0.0001	5	89.7661	<0.0001
$\alpha_i \times \beta_j \times t$	75	1.7383	0.0001	165	4.9837	<0.0001
$\alpha_i \times \beta_j \times t^2$	75	1.3908	0.0175			
$\alpha_i \times \beta_j \times t^3$	75	1.1254	0.2224			

Type III sum of squares was used to assess the model defined in Eq (3-6), where, μ, is the global mean; α_i the age-specific mortality term; β_j the borough or neighborhood term; t , t^2 and t^3 the third order time polynomial; and the double and triple interactions accordingly. These definitions were used at both spatial levels, i. e., borough and neighborhood. In addition, fixed effects, back-step model selection was carried out from the maximal to the current model at the neighborhood level. Empty cells correspond to discarded terms.

by third order time tendency $\beta_j \times t^3$ (empty cell), but, this is not the case at borough level with a $p - value = 0.0177$. With this in mind, the mortality can be pictured at different levels according firstly, to the used model and secondly, to the fixed-effect model term under scrutiny.

The original Fig. 5 (now Fig. 6) panels A, B and C show the obtained posterior Fisher's Least Significance Difference (LSD) test results for α_i, β_j and $\alpha_i \times \beta_j$ fixed-effect model terms respectively, using borough's model estimations. In addition, original Fig. 6 (now Fig. 7) panels C and D shows the LSD neighborhood results for Cuauhtemoc's borough for $\alpha_i|_{Borough=Cuauhtemoc}$ and $\beta_j|_{Borough=Cuauhtemoc}$ fixed-effect model terms respectively. Original Fig. 6 panel B is just a spatial representation of panel D.

Finally, taking about model interpretation, we need to first take a loot at borough's level model. Here, it is clear that the observed mortality can be explained by the different term contribution included in Eq (3- 6) and particularly by the fixed-effect model terms included in Table 1. Particularly, original Fig. 5 showed that when comparing term contributions α_i (age-specific mortality in panel A) against β_j (borough in panel B), the mortality rate is definitely empowered by age-specific groups (upper bounded at $50[x1000]$) rather than spatial borough locations, which is upper bounded at $17[x1000]$. Now, if we look at original Fig. 5 panel C, the $\alpha_i \times \beta_j$, i.e., LSD age-specific times borough model's interaction it can bee seen that the original age-specific mortality rate in panel A is modulated by the borough contribution of panel B. Thus, mean results in panel C by age-specific groups should return into panel's A results. At this level, we are dealing at "between" borough mortality rate level description.

On the other hand, when we turn our attention to the neighborhood modeling level, we are dealing at "within" borough mortality rate data description. In this

context, we have to keep in mind original Fig. 5 panel B mortality rate data order of magnitude, which is going to be fine grained modeled using the same framework depicted in Eq (3- 6). The first result to be discussed is that, unlike borough level, neighborhood mortality rate data does not cope with third order neighborhood interaction and the only triple significant interaction has a linear time tendency for age-specific times neighborhood mortality, as shown in Table 1. The second is the new insight of the mortality rate at the neighborhood level. Here, the first mortality rate level description (at borough) leaves Cuauhtemoc's borough about 15. Now, the original Fig. 6 panels C and D, decomposed into a finer grain considering the same age-specific groups, but, now Cuauhtemoc's neighbors are included, respectively. At this data level description, mortality age-specific groups within Cuauhtemoc are closer to each other (same magnitude order, units) unlike borough level (one magnitude order, tenths). In addition, the inter neighborhood mortality rate is almost shared by all 34 neighborhoods (above 7 and below 8), is we consider the shared letters of the LSD results in original Fig. 6 panel D. Finally, the rest of the mortality rate contribution to sum up to 15 (Cuauhtemoc's borough mortality description), it is distributed upon the different neighborhood model terms of Eq (3- 6) which was neither presented, nor discussed in this manuscript, in order to focus the attention into the principal aspects.

9. I would like to see more discussion on data limitations. How the registry of deaths is assessed, some deaths might be counted in Mexico City even if the individual was not living there simply because there are more hospitals, etc.

Answer: The following paragraph was added in the Discussion section.

> "*Possible intrinsic biases due to possible errors in the registry must be taken into account. For example, the record of a death is registered once a certificate of death has been provided. In some cases, it could last days, depending on diverse factors. However, in the case of Mexico City, that under-registry is extremely low since social and health services are guaranteed in practically the whole area of the city.*"

10. As it is the results section is too focused on the model and less on the actual results. It would also be interesting more discussion on the actual findings. Why is mortality increasing in some parts of Mexico City? Is it because of health care performance? More or less hospitals? health behaviors? etc.

Answer: The following paragraph was added in the discussion section as complementary information of our findings.

> "*Another point to take into account when the mortality rate in Cuauhtémoc is observed, lies on high levels of insecurity for the aforementioned reasons regarding population density and economic concentration. Additionally, the Drug War launched at the end of 2006 by former President Felipe Calderón, affected mortality rates in a large part of the country, being the capital of the country also upset, in particular, the city Downtown, Cuauhtémoc.* "

Minor comments:

1. Line 7. Comparing rates of what?

Answer: The authors have considered the reviewer's suggestion and changed the text accordingly:

Original version MM can be differentiated from rural mortality given the fact that causes and risk factors are substantially different, even when comparing their rates.

Revised version MM can be differentiated from rural mortality given the fact that causes and risk factors are substantially different.

2. Line 12. Do you mean seasonality?

Answer: The authors have considered the reviewer's suggestion and changed the text accordingly:

Original version However, missing data is a common problem in developing and emerging countries, particularly for small-scale spatio-temporal level measurements, such as neighborhood and monthly/seasonal respectively.

Revised version However, missing data is a common problem in developing and emerging countries, particularly for small-scale spatial and temporal level measurements. Due to census limitations and restricted access to public health data, it is challenging to collect information at the neighborhood and monthly level.

3. Line 14. Data types? Do you mean variable?

Answer: The authors have considered the reviewer's suggestion and revised the text accordingly:

Original version One of the most useful data types to explain MM is the age group, since the causes associated to risk factors, disease incidence and other several related variables, are substantially different between such groups.

Revised version One of the most useful variables to explain MM is the age group, since the causes associated to risk factors, disease incidence and other several related variables are substantially different between such groups.

4. Line 18. Please describe which ages are included in each group since the beginning.

Answer: The authors have considered the reviewer's suggestion and revised the nabuscript accordingly:

Original version Often, these age groups are separated into non-overlapping sets as follows: i) Infant, ii) Pre-school, iii) School, iv) Productive and v) Post-productive.

Revised version Often, these age groups are separated into 16 non-overlapping sets as follows: i) Infant ($x < 1$ years old), ii) Pre-school ($1 \leq x < 4$ years old), iii) School ($4 \leq x < 14$ years old), iv) Productive ($14 \leq x < 64$ years old) and v) Post-productive ($x \geq 64$ years old)

5. Please provide a reference to the statement 'To worsen this situation, exposure to environmental risk factors derived from urbanization and migration has increased, with its associated negative health effects gaining attention in recent years.'

Answer: These effects have been thoroughly documented both in the international arena and in the case of Mexico City. Key references in this regard are as follows:

- Cohen AJ, Brauer M, Burnett R, Anderson HR, Frostad J, Estep K, et al. Estimates and 25-year trends of the global burden of disease attributable to ambient air pollution: an analysis of data from the global burden of diseases study 2015. The Lancet 389 (2017) 1907–1918.

- Lelieveld J, Evans JS, Fnais M, Giannadaki D, Pozzer A. The contribution of outdoor air pollution sources to premature mortality on a global scale. Nature 525 (2015) 367
- Bel G, Holst M. Evaluation of the impact of bus rapid transit on air pollution in Mexico City. Transport Policy 63 (2018) 209–220
- Davis LW. Saturday driving restrictions fail to improve air quality in Mexico City. Sci. Rep. 7 (2017) 41652.

6. Line 98. These sentences are not clear.

Answer: The authors have considered the reviewer's suggestion and changed the text accordingly:

Original version Despite it is well known that seasonal variations in environmental factors as well as socio-economical factors, may influence interpretation of the available data for Mexico City, there is no previous report in which time lapses for measurements were taken in smaller scales than in a year time.

Revised version It is known that temporal variations in environmental factors (air pollutants for example) and socio-economical variations (socioeconomic level among boroughs and neigborhoods) can lead to biased results when not having measurements at the appropriate scales. Thus, there is an urgent need to count on fine-grained data in order to better interpret spatio-temporal correlations in health outcomes. At this time there is no previous report in which time lapses for measurements were taken in smaller scales than in a year time and data at neighborhood level can be either scarse or null.

7. Line 115. Please describe since the beginning of the study area section that you refer as Mexico City as what was previously known as D.F. and that it does not include all the Metropolitan Area.

Answer: The authors have considered the reviewer's suggestion and revised the manuscript accordingly:

Original version The study area is the capital of Mexico, Mexico City.

Revised version The study area is the capital of Mexico, Mexico City which was known as the Federal District (Distrito Federal) until 2016. Mexico City belongs to the Valley of Mexico Metropolitan Area, the biggest metropolis in the central region of the country.

8. Line 131. In addition, the global mortality rate group was considered, independent of the age-specific descriptor. This sentence is not clear.

Answer: The authors have considered the reviewer's suggestion and changed the text accordingly:

Original version In addition, the global mortality rate group was considered, independent of the age-specific descriptor

Revised version This sentence was removed as Global mortality is no longer used in the manuscript.

9. Please consider having only one legend in Fig 2. This will allow to see how mortality is high at birth, then decreases and then increases sharply for the post-productive group.

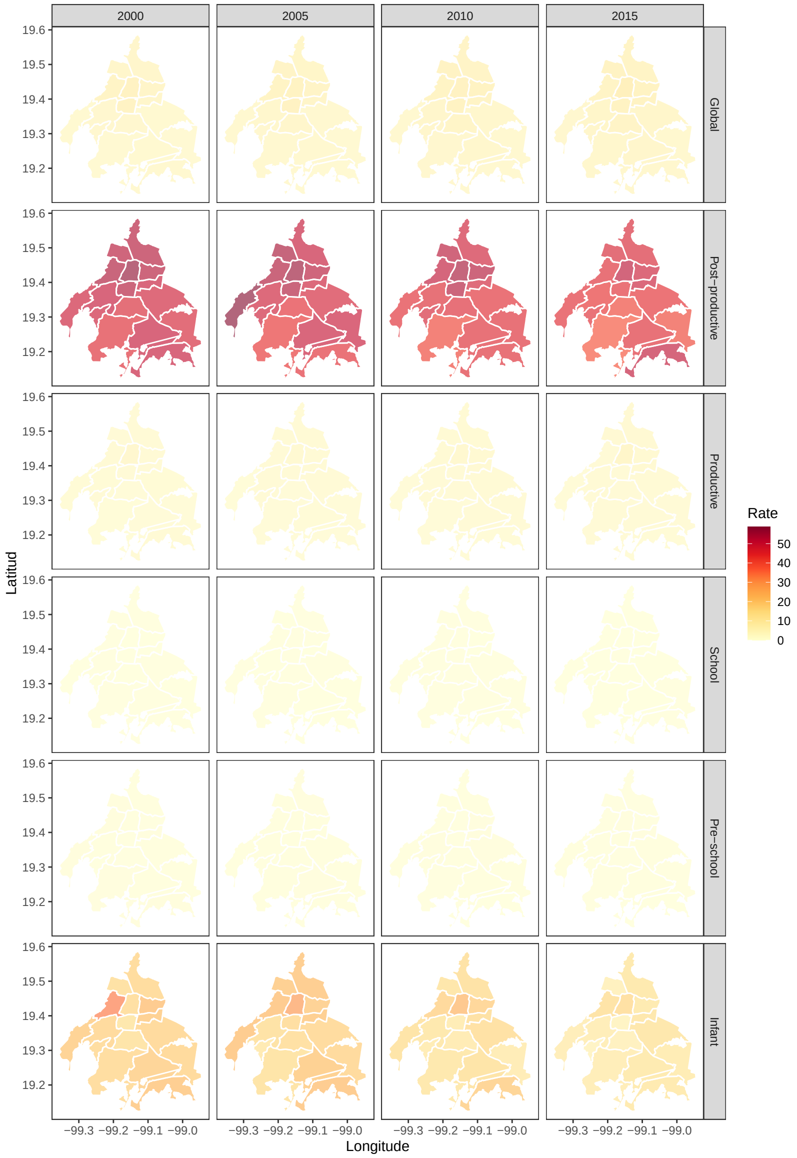

Fig 1. Age-specific mortality rates in Mexico city. Each row corresponds to a particular age-specific mortality rate, i.e., Global, Post-productive, Productive, School, Pre-school and Infant, whereas columns stand for selected years 2000, 2005, 2010 and 2015 from the total period 2000 to 2016.

Answer: Initially the authors considered the reviewer's suggestion, but, as the mortality rate is very different upon the age-specific groups, using a single legend is not informative for borough level as can be depicted in Fig. 1 in this response.

10. Fig 3, consider reducing the size of the x-axis to more clearly see whether rates are going down or up.

Answer: The authors did consider the reviewers suggestion, but, the results would vary according to the selected x-axis time frame considered. Thus, the authors included in the revised version of Fig. 3 a linear fit, across the complete x-axis, to track linear time tendency in order to address the reviewer's suggestion. The manuscript text has been updated in concordance.

11. Fig 4, consider not showing x and y labels.

Answer: The authors have removed x and y labels, according to the reviewer's suggestion.

Reviewer #2

This paper attempts to model metropolitan age-specific mortality at borough and neighborhood level in Mexico City. Because the data is not available at very fine spatial and temporal scales, it uses space-time kriging to get estimates at those scales. The paper then uses a time-evolution mixed effect.

The paper is overall well written, but I question the approach. First, data was only available at the borough level (which is much more coarse than the neighborhood level). Essentially, the authors were trying to fill in the gaps with space-time kriging (which makes sense), but they were trying to 'downscale' to obtain data at the neighborhood level; it seems to me the approach to do that is not correct. Why didn't the authors use a area-to-point kriging? Second, the authors did not provide any estimation of the error for space-time kriging, and their modeling (for instance, a root-mean square error).

Answer: As regards the reviewer's comments to our methodological approach, we can present the following responses:

- Area-to-point (AtP) kriging provides instantaneous estimation of the spatial regression, which is valid for each time point, i.e., it is appropriate for a cross-sectional study, nonetheless, we have both longitudinal and spatial data points.
- In order to overcome the former issue, the authors have considered to use a methodology that considers both temporal and spatial data correlations, falling into spatio-temporal kriging. Under this methodology, the choice of the variance-covariance spatio-temporal structure is not trivial and requires prior knowledge. As far as the authors know, we could not find in the literature reported mortality variance-covariance spatio-temporal structure. Thus, we tackle this issue by parsimony principle using computational force, in order to find the model structure with the least possible error among different mortality variance-covariance spatio-temporal structure available at `gstat` [22, 23] library: metric, separable, productSum, sumMetric and simpleSumMetric. In addition, for each covariance structure, all possible single, double or triple variogram combinations (Exponential, Gaussian and Spherical) were tested, in order to minimize the weighted mean squared error. Complete age-specific mortality tested covariance fitted model results can be found in S1 File Table 2.
- In addition, whenever a time-wise progression estimate is needed, AtP falls short of considering spatio-temporal correlations unless an appropriate time-regression procedure is considered, i.e., one compliant with Gauss-Markov theorem. In this case, a sum-integrated joint method - minimizing error via least squares regression - will give rise to a valid measure; formally a best linear unbiased estimator. Doing this, it will however be formally equivalent to a joint (sumMetric) integrated spatio-temporal estimator, such is the case of our estimators for the different age-specific groups such as Infant (joint=Gaussian), Productive (joint=Exponential) and Post-productive (joint=Gaussian) cases.
- Finally, and as suggested by the reviewer the estimation error for the space-time kriging has been included in the article for the complete age-specific analysed groups ($8.16e - 10$) and in S1 File Table 3, which has been included as Table 2 here for a fast reference.

Table 2. Mortality spatio-temporal kriging age-specific root-mean square error

Mortality age-specific group		Mortality root-mean square error
Infant	($x < 1$ years old)	3.7E-12
Pre-school	($1 \leq x < 4$ years old)	2.7E-15
School	($4 \leq x < 14$ years old)	2.1E-16
Productive	($14 \leq x < 64$ years old)	4.9E-11
Post-productive	($x \leq 64$ years old)	1.8E-09

Additional comments

1. The reader may not understand the term productive and post-productive.

Answer: Here, we refer to the productive group as the economically active population (those in an age bracket that enable them to have jobs) while post-productive is sometimes referred as economically dependant from the productive population.

2. First sentence of the introduction? I disagree with that statement - this is not one of the most challenging. Rather write: 'a challenging'

Answer: The reviewer's suggestion was taken into consideration. The text has been modified as follows: "*Identifying trends in Metropolitan Mortality (MM) is a challenging problem nowadays*"

3. line 10: 'troubles' is not the right word. Also change 'when talking about' . . . this is not a formal way to write.

Answer: The reviewer's suggestion was taken into consideration. The text has been modified as follows: "*However, missing data is a common problem in developing and emerging countries, particularly for small-scale spatial and temporal level measurements. Due to census limitations and restricted access to public health data, it is challenging to collect information at the neighborhood and monthly level.*"

4. line 20: Do you mean 'aggregated' instead of 'collectively'?

Answer: In order to clarify the idea, the text has been modified as follows: "*On the other hand, mortality as well as other health outcomes, can equally be described at different scales, for example, individual and aggregated*".

5. line 31: Suggest you add the reference of Casas et al. (2017), which also uses a multilevel approach.
   - Casas, I., Delmelle, E., & Delmelle, E. C. (2017). Potential versus revealed access to care during a dengue fever outbreak. Journal of Transport & Health, 4, 18-29.

Answer: The reference was added, following the reviewer's suggestion.

6. line 45-50: that paragraph is not clear....need to rephrase

Answer: The authors have considered the reviewer's suggestion and changed the text accordingly:

Original version In the case of developing and emerging countries, it has been described an exacerbated problem in health disparities due to poverty, environmental threats, inadequate access to health care and educational inequalities that may lead to higher mortality rates [24]. Despite these studies rely on partially documented health and socio-demographic registries, it has been possible to gain insights of spatially differences in health outcomes.

Revised version In developing and emerging countries, it has been described an exacerbated problem in health disparities due to poverty, environmental threats, inadequate access to health care and educational inequalities that may lead to higher mortality rates [24]. In most cases, the conducted studies about mortality rates and other health outcomes rely on data that is not well documented or incomplete. Due the lack of economic resources from public institutions or restrictions to this data for research purposes.

7. line 55: seems a stretch to say that air pollution could explain a seasonal pattern in mortality - would be good to have more references to back this up.

Answer: The reviewer is right in their comment. Since seasonal mortality patterns have several varibles (some of them are explained throughout this manuscript), and claiming that one of those variables could explain the seasonal pattern, we decided to modify the Introduction section and eliminate the phrase regarding air pollution as a direct factor of seasonal mortality.

8. line 71: remove 'approach'

Answer: In order to clarify the idea, the text has been modified as follows: "*To overcome data scarcity, Kriging has been used to interpolate spatial missing data at the county level as well as for temporal interpolation in mortality data*"

9. line 75-76: The paper is not about comparing those techniques

Answer: The authors have considered the reviewer's suggestion and changed the text accordingly:

Original version However, despite these studies, there is no consensus about which spatial or spatio-temporal methodology is the best for this purpose.

Revised version However, in current literature, it has not been reported an standard spatio-temporal methodology that properly addresses heterogeneity and data scarcity.

10. It may be useful to add a couple of sentences describing the census in Mexico, like around lines 77.

Answer: We have added a paragraph describing the National Census. The paragraph is as follows:

> "*Regarding the data collection in Mexico, the National Institute of Statistics, Geography and Informatics (INEGI, Instituto Nacional de Estadística, Geografía e Informática) [25], each ten years develops a country-level census of population. In that national survey, economic, demographic and social data of all citizens in Mexico are collected and stored in a public database. Hence, INEGI provides the core of socioeconomic and demographic data, used as input for those databases that we used in this work. In addition, the mortality data was obtained from the Secretariat of Health in Mexico City (Secretaría de Salud de la Ciudad de México: SEDESA) [21]. Detailed description of both databases can be found in the following subsections.*"

11. line 93-94 is an odd sentence - rephrase.

Answer: The authors have considered the reviewer's suggestion and changed the text accordingly:

Original version In Mexico City, health disparities may be enormous between close neighborhoods, even if they belong to the same borough

Revised version In Mexico City, health disparities such as illness and mortality rates can vary between close neighborhoods, just a few blocks apart, within the same borough

12. line 93: 'backwardness' - what is that?

Answer: "Social backwardness" is a term coined to refer to the lack of advancement of a group relative to the average.

13. line 96: change 'arise' to 'arises'

Answer: In order to clarify the idea, the text has been modified as follows: "*A similar behaviour arises if we consider the temporal component: coarse-grained measurements may mask the variant behaviour of MM*"

14. sentence on line 98-101 is awkward. Rephrase.

Answer: The authors have considered the reviewer's suggestion and changed the text accordingly:

Original version Despite it is well known that seasonal variations in environmental factors as well as socio-economical factors, may influence interpretation of the available data for Mexico City, there is no previous report in which time lapses for measurements were taken in smaller scales than in a year time.

Revised version It is known that temporal variations in environmental factors (air pollutants for example) and socio-economical variations (socioeconomic level among boroughs and neigborhoods) can lead to biased results when not having measurements at the appropriate scales. Thus, there is an urgent need to count on fine-grained data in order to better interpret spatio-temporal correlations in health outcomes. At this time there is no previous report in which time lapses for measurements were taken in smaller scales than in a year time and data at neighborhood level can be either scarse or null.

15. line 110 - remove 'the' before 'MM'

Answer: In order to clarify the idea, the text has been modified as follows: "*Thus, a finer description level of these phenomena may result in a more accurate spatio-temporal description of MM in Mexico City.*"

16. Fig 1 - add an inset map

Answer: A mini-map of Mexico, with Mexico City (in red), was included in Panel A of Fig. 1 as suggested by the reviewer.

17. mortality database - is the data available at neighborhood level? I guess not, and that is the point of kriging...although as I mentioned, another technique should be used

Answer: The mortality database is available only at borough level, thus, not available at the neighborhood level as the reviewer pointed out. That is why the authors h uavesed Kriging in order to fill the gaps at the neighborhood level, starting from available borough data points. In our approach we used two, hierarchical approach first with ordinary Kriging and then with two independent linear mixed-effects models to address the modifiable areal unit problem (MAUP) about the two spatio-temporal scales (borough and neighborhood), instead of using area to point kriging as recommended by the reviewer.

18. We don't know the root mean square error for kriging estimates. Was a cross-validation used?

Answer: Ordinary Kriging was used for each age-specific mortality rate estimation, using all the available data points, under the best covariance model description (metric, separable, productSum, sumMetric or simpleSumMetric), with the best single, double or triple variogram combinations (Exponential, Gaussian and Spherical) according to the minimum weighted mean squared error (see, S1 File Table 2). The neighborhoods centroids at a trimester level, in the period 2000 to 2016 were used to obtain the corresponding each age-specific mortality rate estimation. In this context, the authors took advantage of the best variance-covariance spatio-temporal structure to modulate all the available data point contribution at the new mortality data point estimation. Finally, as suggested by the reviewer the estimation error for the space-time kriging has been included in the manuscript for the complete age-specific analysed groups ($8.16e-10$), and in detailed for every age-specific mortality group in S1 File Table 3, which has also been included as Table 2 in this document, for a fast reference.

References

1. Kwok OM, Underhill AT, Berry JW, Luo W, Elliott TR, Yoon M. Analyzing longitudinal data with multilevel models: an example with individuals living with lower extremity intra-articular fractures. Rehabilitation psychology. 2008;53(3):370.
2. Raudenbush S, Bryk AS. A hierarchical model for studying school effects. Sociology of education. 1986; p. 1–17.
3. Zhang W, Zhang M. Short-and long-term effects of land use on reducing personal vehicle miles of travel: Longitudinal multilevel analysis in Austin, Texas. Transportation Research Record. 2015;2500(1):102–109.
4. Anaya G, Al-Delaimy WK. Effect of the US-Mexico border region in cardiovascular mortality: ecological time trend analysis of Mexican border and non-border municipalities from 1998 to 2012. BMC public health. 2017;17(1):400.
5. Nesterko Y, Turrión CM, Friedrich M, Glaesmer H. Trajectories of health-related quality of life in immigrants and non-immigrants in Germany: a population-based longitudinal study. International journal of public health. 2019;64(1):49–58.
6. Dolev T, Zilcha-Mano S. The role of the therapeutic relationship in the association between interpersonal behaviors and outcome: Comparison of two competing models. Psychotherapy Research. 2019;29(5):553–564.
7. Green EP, Cho H, Gallis J, Puffer ES. The impact of school support on depression among adolescent orphans: a cluster-randomized trial in Kenya. Journal of Child Psychology and Psychiatry. 2019;60(1):54–62.

8. R Core Team. R: A Language and Environment for Statistical Computing; 2018. Available from: https://www.R-project.org/.

9. Pebesma E. Simple Features for R: Standardized Support for Spatial Vector Data. The R Journal. 2018;10(1):439–446. doi:10.32614/RJ-2018-009.

10. Bivand R, Rundel C. rgeos: Interface to Geometry Engine - Open Source ('GEOS'); 2018. Available from: https://CRAN.R-project.org/package=rgeos.

11. Hijmans RJ. raster: Geographic Data Analysis and Modeling; 2019. Available from: https://CRAN.R-project.org/package=raster.

12. Hijmans RJ. geosphere: Spherical Trigonometry; 2017. Available from: https://CRAN.R-project.org/package=geosphere.

13. Pebesma E. spacetime: Spatio-Temporal Data in R. Journal of Statistical Software. 2012;51(7):1–30.

14. Pebesma EJ, Bivand RS. Classes and methods for spatial data in R. R News. 2005;5(2):9–13.

15. Bivand R, Keitt T, Rowlingson B. rgdal: Bindings for the 'Geospatial' Data Abstraction Library; 2019. Available from: https://CRAN.R-project.org/package=rgdal.

16. Wickham H. ggplot2: Elegant Graphics for Data Analysis. Springer-Verlag New York; 2016. Available from: http://ggplot2.org.

17. Wilke CO. cowplot: Streamlined Plot Theme and Plot Annotations for 'ggplot2'; 2019. Available from: https://CRAN.R-project.org/package=cowplot.

18. Auguie B. gridExtra: Miscellaneous Functions for "Grid" Graphics; 2017. Available from: https://CRAN.R-project.org/package=gridExtra.

19. Dunnington D. ggspatial: Spatial Data Framework for ggplot2; 2018. Available from: https://CRAN.R-project.org/package=ggspatial.

20. Di Rienzo J, Casanoves F, Balzarini M, Gonzalez L, Tablada M, Robledo C. InfoStat; 2011.

21. Secretaría de Salud de la Ciudad de México. Secretaría de Salud de la Ciudad de México; 2019. http://data.salud.cdmx.gob.mx/portal/.

22. Pebesma EJ. Multivariable geostatistics in S: the gstat package. Computers & Geosciences. 2004;30:683–691.

23. Gräler B, Pebesma E, Heuvelink G. Spatio-Temporal Interpolation using gstat. The R Journal. 2016;8:204–218.

24. Ayele DG, Zewotir TT. Childhood mortality spatial distribution in Ethiopia. J Appl Stat. 2016;43(15):2813–2828.

25. Instituto Nacional de Estadística y Geografía. Instituto Nacional de Estadística y Geografía; 2019. https://www.inegi.org.mx/.
