# Supplementary material for: Metropolitan age-specific mortality trends at borough and neighborhood level: The case of Mexico City
Source: PLoS One. 2021 Jan 19;16(1):e0244384. doi: 10.1371/journal.pone.0244384 (PMC7815139; doi:10.1371/journal.pone.0244384)
Supplement: Attachment — Submitted filename: Response to Reviewers PONE-D-20-10411R1.pdf [file pone.0244384.s004.pdf]

Response to Reviewers

PONE-D-20-10411R1

Metropolitan age-specific mortality trends at borough and neighbourhood level: The case of Mexico City

PLOS ONE

Dear Professor Zhang, and Reviewers: Please find our detailed response to your editorial assessment enclosed in the present document. To ease readability our responses appear in **bold blue print**.

The authors are grateful to the Academic Editors and the Reviewers for their suggestions and critique of our work.

Additional Editor Comments (if provided):

Related to my previous comments, the authors provide informative responses and in-depth discussions. I appreciate their efforts. But **I would suggest that they should provide a succinct discussion at the end of the paper in response to the MAUP issue (my previous comment #3) and modeling selection (my previous comment #4), by discussing existing methods and literature.** Please do make some improvements before it can be acceptable. This can add two points of value to your paper. First, readers can be benefited from knowing how you select your methods (by comparing other similar methods) and how do you think about the MAUP issue. Second, it will make your paper more self-reflective by discussing some limitations or future work.

We agree with the Editor's suggestion about addressing the MAUP in the manuscript. This discussion is now included in the revised version of the manuscript.

Since Reviewer #2 did not provide a further review at the sound round, I take over his role and would like the authors to address more when responding to Reviewer #2's comments, as follows:

1. I think, like above, the authors provided a good response to Comment #1. But **no modification or additional information are provided in the manuscript for addressing the issues. If the reviewer had this question, possibly some readers would have similar queries**.

2. Similarly, do check whether you've **clarify those issues in Comments #1, 12 in your manuscript, rather than only in the response letter**.

We agree with the Editor's suggestion about addressing these methodological issues also in the manuscript. This discussion is now included in the revised version of the manuscript.

Reviewers' comments:

Comments to the Author

Reviewer #1: The authors have addressed my concerns from my previous review. I have only minor changes to address in this second round of review:

Small comments:

The sentence 'Individual mortality is influenced by personal-level characteristics such as genetics, socioeconomic status and education' **needs a reference.**

References have been provided to support for this statement

The sentence 'To worsen this situation, exposure to environmental risk factors derived from urbanization and migration has increased, with its associated negative health effects gaining attention in recent years' is backed up with references about air pollution and urbanization, but there is no support for migration worsening health, in fact in research the evidence

of this is mixed, with some cases having even a positive impact on population health. **I suggest to not refer to migration as a risk factor.**

We have re-worded this section for clarity. We have also eliminated the reference to migration as a risk factor, as the reviewer suggested.

In the references, **the correct name of INEGI is Instituto Nacional de Estadística y Geografía.**

We have corrected this in the revised version of the manuscript.
